# Editorial: Biological therapy for allergic diseases: peculiarities, prospects, and challenges

**DOI:** 10.3389/falgy.2024.1440549

**Published:** 2024-07-10

**Authors:** Ervin Ç. Mingomataj, Tayseer Ibrahim, Syed A. Rizvi

**Affiliations:** ^1^ Department of Allergology & Clinical Immunology, “Mother Theresa” School of Medicine, Tirana, Albania; ^2^ Allergy and Immunology Division, Hamad Medical Corporation, Doha, Qatar; ^3^ College of Biomedical Sciences, Larkin University, Miami, FL, United States

**Keywords:** allergic diseases, biologics, COVID-19, genome-wide association studies, immunology, monoclonal antibodies, panitumumab, targeted personalized therapy

**Editorial on the Research Topic** Biological therapy for allergic diseases: peculiarities, prospects, and challenges

Understanding the phenotypes and endotypes of immuno-allergic diseases has facilitated biologics’ use as a targeted and stratified treatment approach. Despite the documented effectiveness, the heterogeneous nature of these diseases represents a continuous challenge for this treatment. Thus, the section of Therapies, Therapeutic Targets & Mechanisms of the *Frontiers in Allergy* launched the Research Topic *Biological Therapy for Allergic Diseases: Peculiarities, Prospects, and Challenges* that compiles two review and original research articles each, one data report and one case report on new, interesting aspects, all centered around the mentioned topic.

A literature survey provided by Lombardi et al. studied the role of different biological agents (omalizumab, mepolizumab, benralizumab, reslizumab, dupilumab, etc.) among severe asthma patients in the COVID-19 era. Analyzing data from 15 studies, these authors did not find their use as a risk factor for asthma aggravation among COVID-19 comorbid patients (in contrast to patients with additional pathologies such as diabetes, obesity, and systemic hypertension), suggesting, therefore, continuing this treatment in such cases with special attention to the mentioned comorbidities. Moreover, they found out that therapeutic use of anti-interleukin 5 antibodies (like mepolizumab) does not result in complete suppression of blood and bone marrow eosinophil levels, allowing an eosinophil-depended protective effect to SARS-CoV-2 infection.

A Mendelian randomization study conducted by Li et al. using genome-wide association studies datasets discovered potential causal relationships between specific inflammatory cytokines and pathologic nasal conditions, including allergic rhinitis (AR), nasal polyps (NP), etc. According to this investigation, elevated circulating levels of macrophage inflammatory protein MIP-1*α* and tumor necrosis factor TNF-α were consistent risk factors for the AR, and an increased level of circulating interleukin IL-2 was a susceptible risk factor to NP. In contrast, elevated circulating platelet-derived growth factor PDGF-BB levels revealed a decreased NP risk. Admitting that Mendelian randomization can only provide potential insights into causality, these authors stated that a better functional understanding of these inflammatory factors in the pathogenesis of these diseases could offer new possibilities for generating more effective therapeutic strategies (such as synthesis of specific biologicals).

The paper by Sitek et al. shows a comprehensive review of hypersensitivity reactions associated with monoclonal antibodies used to treat allergic disorders. It referenced several FDA-approved monoclonal antibodies for conditions such as asthma, chronic spontaneous urticaria, chronic rhinosinusitis with nasal polyps, hypereosinophilic syndrome, atopic dermatitis, and eosinophilic esophagitis. While these biologics offer significant benefits, they can also lead to hypersensitivity reactions, categorized as immediate (including anaphylaxis) and delayed (such as serum sickness reactions and type IV reactions). The paper discussed clinical features, diagnostic methods like skin testing, and management strategies, including desensitization protocols. It highlights the need for more research to address knowledge gaps and improve the diagnosis and management of hypersensitivity reactions to these biologics.

Esposito et al. explored the autoimmune aspects of severe eosinophilic asthma (SEA) by investigating autoantibodies and their targets. They tested for autoantibodies against proteins involved in oxidative post-translational modification (oxPTM) in SEA patients and compared them to those in patients with eosinophilic granulomatosis with polyangiitis (EGPA) and healthy controls. They used immunofluorescence and ELISA techniques to detect these autoantibodies. The results showed that some SEA patients had autoantibodies against specific proteins like MPO, Collagen-V, TREM1, and IL1R2, although none of these proteins alone demonstrated high sensitivity for SEA. Interestingly, the proportion of SEA patients with positive autoantibodies was higher than healthy controls. However, the study did not reveal a significant difference between autoantibodies against native and oxPTM proteins. While no single protein marker was highly indicative of SEA, the presence of autoantibodies in a large SEA patient proportion suggests the potential for further research into autoantibody serology to improve diagnostic testing for severe asthma.

Understanding the pathophysiology of allergic diseases facilitates the development of targeted, personalized therapy. Monoclonal antibodies that interfere with type 2 inflammatory immune response and IgE are used to treat various allergic diseases, in addition to allergen immunotherapy (AIT), which has been proven effective and a potential disease-modifying treatment. Many approaches were proposed to decrease the duration of AIT and its side effects; one of them is biological therapy alone or in conjunction with AIT. Atanasio et al. outlined the inflammatory response cascade in type 2 inflammation and IgE production. They highlighted the mechanism of action of allergen-specific monoclonal antibodies and type 2 cytokines monoclonal antibodies (epithelial-derived cytokines, IL4Ra, and anti-IgE biologics). The review referenced multiple studies that reported the effect of these biologicals on allergic disease-specific symptoms and/or AIT side effects. It also emphasized the challenges and called for further research to address the unmet needs in this subject.

The use of type 2 monoclonal antibodies, originally used for treating allergic diseases, was reported by Konstantinou and Voukelatou for treating an eosinophilic condition other than allergic disease. They presented a patient diagnosed with eosinophilic cystitis according to clinical history, cystoscopy findings, and histopathological examination. Treatment with corticosteroids was unsuccessful, and the patient was unfit for surgical intervention, which is the second-line management. A 12-month treatment with benralizumab, an anti-IL5R monoclonal antibody, has led to complete resolution of symptoms and histopathological changes; disease resolution was sustained even after discontinuation of benralizumab treatment.

The Research Topic *Biological Therapy for Allergic Diseases: Peculiarities, Prospects, and Challenges* represents an inspiring and informative article collection, as shown in this summary.

